# Is Isolated Inferior Rectus Weakness a Strong Indicator for Myasthenia Gravis?

**DOI:** 10.22599/bioj.502

**Published:** 2026-04-02

**Authors:** Emma Butterworth, Jessy Choi, Martin Rhodes, Joshua Simmons

**Affiliations:** 1 Sheffield Teaching Hospitals NHS Foundation Trust, UK

**Keywords:** Myasthenia Gravis, MG, Inferior Rectus, Acetylcholine receptor antibody, Acetylcholine receptor antibody testing

## Abstract

**Background:**

Isolated inferior rectus weakness (IRW) has historically been reported as a possible presenting sign of myasthenia gravis (MG). Orthoptists often identify IRW, prompting further investigations. This service evaluation aims to determine whether patients with IRW or other symptoms were subsequently diagnosed with MG.

**Methods:**

A retrospective review of patient case notes was conducted. The cohort included consecutive patients who presented to the adult strabismus service and received acetylcholine receptor antibody (ACR) testing between 2010 and 2023 at a tertiary referral centre in Sheffield.

**Results:**

Sixty-two patients were included in the analysis. Patients were grouped based on isolated IRW, suspicion of thyroid eye disease, or the presence of MG characteristics. These characteristics included variable diplopia, ocular motility and ptosis; fatigue on elevation; Cogan’s lid twitch; MG-like fatigue; and breathing or swallowing difficulties.

Ten patients (16.13%) were diagnosed with MG. Of these, six (60%) had ocular MG (OMG). No patients in the isolated IRW group were found to have MG. All MG patients exhibited weakness of multiple extra-ocular muscles (EOM), ranging from 2 to 12 different muscles affected. Each had one or more characteristic MG symptoms or signs, with variability observed in 80% (n = 8).

**Conclusion:**

No cases of MG were identified in patients with isolated IRW. All MG diagnoses were associated with characteristic signs or symptoms. This highlights the importance of detailed clinical history and orthoptic assessment when MG is suspected and poses further questions of whether isolated IRW needs further MG investigative testing.

## Introduction

Myasthenia gravis (MG) is an autoimmune disease occurring within the neuromuscular junction of predominantly oculobulbar muscles (Deymeer, 2020). If only ocular muscles are affected, such as the extra-ocular muscles (EOM), levator, and orbicularis, it is diagnosed as ocular MG (OMG). Whereas, if additional or other non-ocular muscles are affected, it is diagnosed as generalised MG (GMG) (Gilhus and Verschuuren, 2015). MG pathophysiology involves the production of autoantibodies targeting the motor end plate (Behbehani, 2023). This results in various and variable symptoms such as diplopia, ptosis, ocular motility weakness, fatigue, breathing difficulties, swallowing difficulties, facial weakness, limb weakness and neck weakness (Shuey, 2022; Uzawa *et al*., 2020). The prevalence of MG has been quantified as between 3 and 30 per 100,000 (Avidan *et al*., 2014). Incidence varies depending upon age and gender, with females more commonly presenting earlier in life compared to males (Zach *et al*., 2013).

MG can remain ocular; however, there is a risk that it can progress to GMG. This risk is greatest within the first two years of initial presentation (Gilhus and Verschuuren, 2015). Rates of progression from OMG to GMG have varied between studies, with a range of 23.6–85% (Gengadharan *et al*., 2024; Nagia *et al*., 2015; Hendricks *et al*., 2019). Patients with OMG and higher detectable ACR levels have been found to have a higher risk of progressing to GMG (Gengadharan *et al*., 2024).

Co-occurring autoimmune conditions may be present with MG, with thyroid dysfunction being the most common (Shabto *et al*., 2024). Thyroid eye disease can present with ocular motility restrictions that mimic OMG (Shabto *et al*., 2024). Therefore, thyroid function tests are recommended for patients with or suspected of having MG (O’Hare and Doughty, 2019). At presentation, up to 22–40 % of OMG patients may also have thyroid dysfunction (Gengadharan *et al*., 2024; Shabto *et al*., 2024).

MG carries a mortality risk. The average mortality rate of MG has been found to be 1.4 cases per million people per year from a recent systematic review with the risk being higher in males (Zhang *et al*., 2023; Sciancalepore *et al*., 2024).

### Ocular characteristics of MG

Ocular symptoms and manifestations of MG include ptosis, diplopia and ocular motility defects with fluctuation related to fatigue. These can be the initial presenting symptoms and signs of MG in 50–60% of cases (Gilhus and Verschuuren, 2015). The EOM generally have fewer acetylcholine receptors and as they are singly innervated, it is thought that less acetylcholine is transmitted across the neuromuscular junction making them more susceptible to autoantibodies. However, EOM specific research is hindered by the size of the muscle in comparison to other muscle groups (Nair *et al*., 2014; Cetin *et al*., 2020).

### Acetylcholine receptor antibody (ACR) testing

The sensitivity of positive ACR in OMG has varied between studies; a recent retrospective cohort of 114 patients found it to be 80% (Chung *et al*., 2021). Whereas prior to this, positive ACR has been found in 40–77% of patients with OMG compared to 80–99% of GMG patients (Gengadharan *et al*., 2024; Nair *et al*., 2014; Cetin *et al*., 2020).

The diagnosis of MG is not based solely on ACR testing. MG can occur in the absence of ACR when characteristic symptoms and signs are present, together with additional investigations. These include single-fibre EMG, which is the gold-standard diagnostic test, or a positive response to a cholinesterase inhibitor such as pyridostigmine, arranged by the neurology department (Ding *et al*., 2020; Li *et al*., 2018; Teo *et al*., 2018). A study of pyridostigmine on OMG patients showed improvement in 97.8% of patients (Gengadharan *et al*., 2024).

### Isolated inferior rectus weakness (IRW)

Historic single and small sample case reports have indicated that isolated IRW can lead to the diagnosis of MG without any additional symptoms (Goyal and Pless, 2009; Garnham and Lee, 2009; Choi *et al*., 2012; Yadav *et al*., 2021). Literature has varied in the reported additional symptoms and incidences of MG of patients presenting with isolated IRW. A review of all isolated IR palsy literature from 1974–2006 found MG occurred in 4 out of 49 cases (8.2%) (Goyal and Pless, 2009). Whereas in a retrospective review of 14 suspected MG cases with an initial presentation of IRW, additional symptoms were also present (Garnham and Lee, 2009). The additional symptoms included lid involvement such as ptosis. This indicated that the IRW was not isolated in the vast majority in the published cohort, with additional symptoms reported to be present in 92.86% (Garnham and Lee, 2009). In another cohort which aimed to retrospectively review aetiology of IRW, 5 patients (11%) with IRW were found to have MG. However, there was no documentation of characteristic MG symptoms or signs such as breathing, swallowing difficulties, lid involvement or diplopia (Choi *et al*., 2012). Similarly, there is also a single case report of IR paresis or paralysis with the patient being diagnosed with MG but additional characteristics of MG were also present (Yadav *et al*., 2021).

Due to these historic reports, the link of isolated IRW to MG has been embedded within Orthoptist’s training and emphasised on its specific associated to MG, over and above all other EOM. This can often lead to targeted attention towards even minor isolated IRW and initiate discussions for immediate investigation of MG. This may occur regardless of the presence or absence of other clinical features or evidence of MG. Alongside this, there may be an explanation for the isolated IRW, such as previous ocular history, including strabismus surgery. In addition to this, anecdotally, when patients with isolated IRW have been tested for MG by the authors, they were rarely found to have the condition. Further exploration of this area was warranted to see if further investigation of patients with isolated IRW for MG is clinically required.

The primary objective of this service evaluation was to determine how many patients within the cohort (adults within the strabismus service who had received ACR testing) with isolated IRW had MG. The secondary objective was to compare the symptoms and signs of patients with MG and to identify those that occur most commonly, particularly those that may be highlighted during an Orthoptic assessment. The specific interest was in whether IRW occurred and whether its presence was significant, compared to other EOM in patients with MG.

## Methods

A retrospective case notes review of consecutive patients presenting to Sheffield Teaching Hospitals NHS Foundation Trust adult strabismus service that had ACR testing requested by strabismus consultants, which was a standard first-line investigation, between 23/06/2010 and 15/08/2023. Patients were identified using the integrated clinical environment (ICE) system. Ethical approval was not required as this was a service evaluation; however, local trust approval and registration were carried out (Clinical Effectiveness Unit reference: 11829).

**Figure 1:**
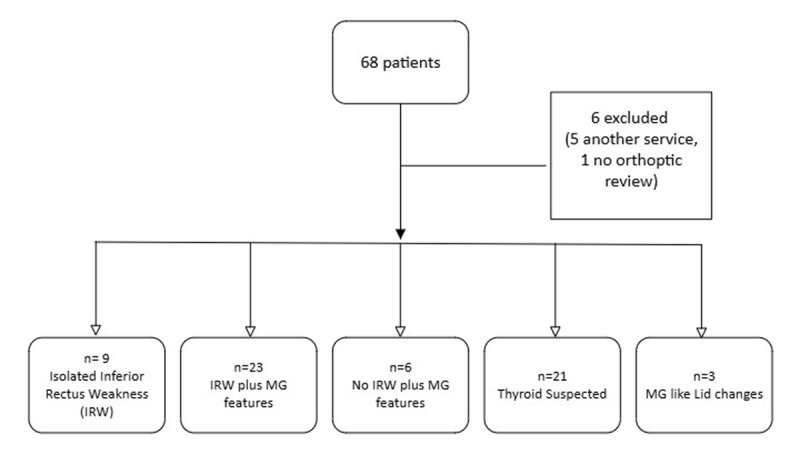
Flowchart of patients included for analysis and subgroups of characteristic MG symptoms and signs. MG features included one or more of: variable diplopia, fluctuating ocular motility and ptosis; fatigue on elevation; Cogans lid twitch; tiredness; breathing and/or swallowing difficulties.

Patient notes were reviewed in either paper format or via the electronic document management system (EDMS). Standardised information was obtained and analysed using an encrypted Excel spreadsheet using non-identifiable patient information. To meet the objectives of this cohort study, each patient was grouped depending on characteristic MG symptoms or signs prior to ACR testing (Figure 1). Features are defined as one or more of the following: variable diplopia with fluctuating ocular motility and ptosis; fatigue on elevation; Cogan’s lid twitch; tiredness; breathing and/or swallowing difficulties. Two additional sole groups were formed, firstly due to the prevalence of thyroid eye disease with MG and secondly, during data collection, it was found that some patients had ACR testing requested purely due to MG-like lid changes without any additional features. This gave a total of 5 individual groups: isolated IRW; IRW plus MG features; no IRW plus MG features; thyroid suspected; and MG-like lid changes without any other MG features.

## Results

Sixty-eight patients were identified over the specified period. Of these, six were excluded; five due to not being managed within the adult strabismus service and one patient had an incomplete data set due to no orthoptic assessment. The remaining 62 were included in analysis. The mean age at presentation for all patients included in analysis was 60.05 (range 20–88) years, with 61% (n = 38) male.

MG was confirmed in 16.13% (n = 10) of the cohort. The diagnosis of MG was made by the neuro-ophthalmology or neurology department following referral from the strabismus service. MG diagnosis was based on symptoms and clinical signs, ACR test result and other diagnostic testing such as anti-muscle-specific kinase or single fibre electromyography and/or with demonstrable improvement following treatment trial with pyridostigmine.

**Figure 2:**
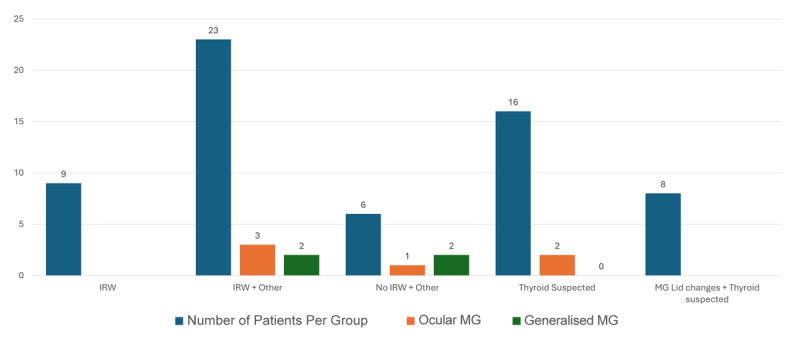
Number of patients in each subgroup shown by the blue bars; number of patients in each subgroup who were diagnosed with ocular MG (OMG) shown by the orange bars; number of patients in each subgroup who were diagnosed with generalised MG (GMG).

Out of the MG patients, OMG was diagnosed in 60% (n = 6) and the remaining 40% (n = 4) with GMG. Of the MG patients, 50% (n = 5) were within the IRW plus MG features group; 30% (n = 3) in no IRW plus MG features group and 20% (n = 2) in the thyroid suspected group were diagnosed with MG (Figure 2). No patients with isolated IRW were diagnosed with MG and they were all ACR negative.

Specific demographics for MG patients included 80% (n = 8) of patients were male. The average age for MG positive males was 66 years (range 34–81), whereas the average age for females was 46.5 years (range 22–71).

All patients had ACR testing, but in view of no patients being diagnosed with MG in the isolated IRW group further exploration occurred to determine if a specific aetiology could be identified. At the time of assessment, all patients underwent a comprehensive orthoptic investigation. As isolated IRW was apparent, further investigative testing was requested in line with current practise which indicated MG should be suspected in isolated IRW. Within the isolated IRW patients’ group, 55.6% (n = 5) had previous strabismus surgery involving either a horizontal or vertical procedure. Other ocular history included an acoustic neuroma, restricted eye movements which did not correlate to a cranial nerve palsy and dry eye disease which occurred in 33.3% (n = 3). The remaining 11.1% (n = 1) had a history of trauma.

**Figure 3:**
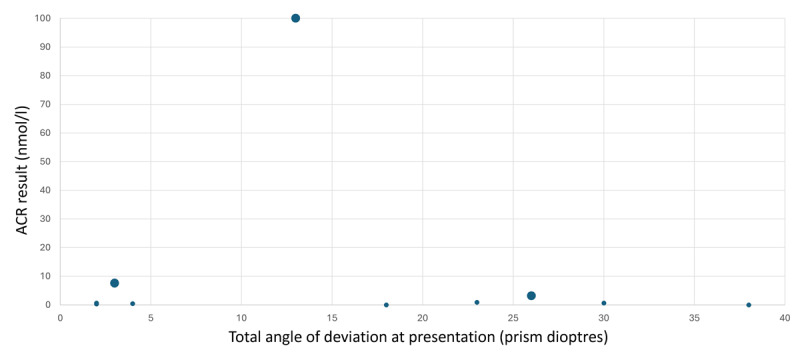
Comparison of total distance angle at presentation measured in prism dioptres and ACR results of positive MG patients measured in nano-moles per litre. OMG is indicated by the larger circle. Notably, two patients had the same angle of deviation and ACR level (0.4 nmol/L); one of these patients had GMG and the other had OMG.

ACR levels varied among the MG-positive patients, ranging from 0 to greater than 100 nmol/L, with a mean of 9.58 nmol/L (Figure 3). A positive ACR result given from the testing centre was considered as 0.2 nmol/l and above. Across all groups, of the MG positive patients, 80% (n = 8) had detectable ACR. To assess whether there was any correlation between the angle of deviation at presentation and ACR levels, the results of both variables were compared. As shown in Figure 3, no correlation was observed. Twenty percent (n = 2) of MG-positive patients did not have detectable ACR; both patients had generalised myasthenia gravis (GMG). As shown in Figure 3, differences in ACR levels between GMG and ocular myasthenia gravis (OMG) are illustrated by the larger circles representing OMG patients. Notably, two patients had the same angle of deviation and ACR level (0.4 nmol/L); one of these patients had GMG and the other had OMG.

All patients with MG exhibited weakness or restriction in two or more EOMs (range 2–12) with an average of seven EOMs prior to MG diagnosis. The superior rectus (SR) muscle was the most frequently affected, occurring in 22.9%, while the superior oblique (SO) was the least affected, in 8.6%. The remaining muscles were all similarly affected. This included the inferior oblique (IO) in 18.6%, both the lateral (LR) and medial rectus (MR) in 17.1%, and the inferior rectus (IR) in 15.7%. As multiple muscles were affected in each patient, the total number of patients affected by each muscle is as follows: SR, nine patients; IO, eight patients; LR, six patients; MR, seven patients; IR, eight patients; and SO, six patients.

**Figure 4:**
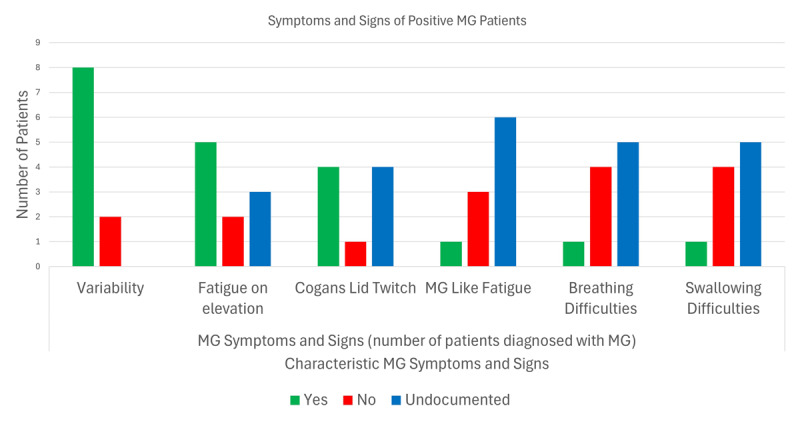
Characteristic MG symptoms and signs present in positive MG patients. Green bar indicates present, red bar indicates not present, and blue bar indicates not documented.

Symptoms and signs of positive MG patients were reviewed. All MG patients had one or more of the following: Variability of ptosis, diplopia, ocular motility or ocular misalignment was documented in 80% (n = 8) of patients, followed by fatigue on elevation in 50% (n = 5) and 40% (n = 4) who had Cogan’s lid twitch. Fatigue associated with MG and breathing and swallowing difficulties were present in 10% (n = 1) of MG positive patients. Due to the retrospective nature of our service evaluation, some symptoms and signs were not available (Figure 4).

Pyridostigmine was given to all confirmed MG patients after a subsequent referral to the neurology service. An improvement in symptoms was seen in 50% (n = 5), an unknown response in 30% (n = 3) and no response in 20% (n = 2). It is unclear whether the lack of response to pyridostigmine was due to the patients not having MG or another reason.

Sadly, one patient passed away. Despite rapid investigation, diagnosis, and treatment, the cause of death was aspiration pneumonia. There was a weakness of six EOM prior to ACR testing. All typical MG symptoms and signs were present which included: variability, fatigue on elevation, Cogan’s lid twitch, MG like fatigue, breathing and swallowing difficulties. The ACR was negative at a value of 0 nmol/l at referral to neurology. The diagnosis was based on the presence of typical signs and symptoms of MG and good clinical response to treatment.

## Discussion

This service evaluation of adults who underwent ACR testing for suspected MG aimed to investigate the presence and relevance of an isolated IRW. Ten (16.13%) patients in this cohort received a diagnosis of MG. The groups with MG diagnoses were isolated IRW plus MG features and no IRW plus MG features and thyroid suspected. No patients in the isolated IRW group were diagnosed with MG. The average age for males with MG in this cohort was 66 years (range 34–81) compared to the average age for females, which was 46.5 years (range 22–71). This has also been found in previous studies (Shuey, 2022).

In the thyroid suspect group, 9.52% (n = 2) were found to have OMG. This figure corresponds to 33.3% of the OMG cases. This aligns with previous findings of 22–40% of patients with thyroid dysfunction having OMG (Gengadharan *et al*., 2024; Shabto *et al*., 2024). Both patients had additional characteristic MG symptoms or signs, which were variability, Cogan’s, and fatigue on elevation.

All patients diagnosed with MG had characteristic symptoms and signs present. This mirrors findings by Garnham and Lee (2009), who found patients with MG also had an additional characteristic MG symptom of ptosis in 92.9% of their cohort. This leads to further discussion on the need for investigation of patients with isolated IRW with no other systemic or ocular characteristics of MG. Even with exclusion of patients who had previous strabismus surgery, this observational cohort did not find any patients with isolated IRWs to be diagnosed with MG.

A positive response from pyridostigmine was demonstrated in 50% (n = 5) of patients diagnosed with MG; however, records were not available for 30% (n = 3), therefore this may be an underestimate. A further 20% of patients (n = 2) showed no change after being prescribed pyridostigmine, with suboptimal compliance related to intolerance to side effects. In our cohort, 10% (n = 1) of positive MG patients passed away from aspiration pneumonia. MG patients are more at risk of developing pneumonia (Su *et al*., 2022). This highlights the mortality risk of MG.

Findings from this service evaluation show the importance of a full and comprehensive case history in any MG suspect along with ensuring clinical signs are assessed. It is important for orthoptists to discuss with the patient if they have any GMG-specific features, such as fatigue, breathing and swallowing difficulties, in addition to an assessment of their eye movements. Assessments should include a detailed ocular motility assessment, an assessment of lid involvement, and a careful comparison between visits. It is important that the method of this testing is standardised amongst clinicians along with responses of subjective tests. For example, when assessing lid involvement in suspected MG patients, positive signs may include Cogan’s lid twitch, fatigue on elevation, and enhanced ptosis. Cogan’s lid twitch is assessed by asking the patient to look downward for 10–15 seconds before quickly returning to the primary position. A positive sign is an upward twitch of the affected eye(s) before returning to its original position (Pietris *et al*., 2023). Fatigue on elevation is positive when ptosis worsens or varies after prolonged elevation for approximately 60 seconds, which is due to the involvement of the affected levator palpebrae superioris (Shuey, 2022; Nair *et al*., 2014). Enhanced ptosis, or the curtain’s sign, is positive when the ptotic lid is lifted and ptosis is seen or worsens in the other eye due to Hering’s law, as yoke muscles receive equal innervation (Behbehani, 2023).

It may also be helpful to include additional tests such as ice pack testing (Uzawa *et al*., 2020). The ice pack test may be performed in clinic by applying it to the ptotic eye for between 3–5 minutes; a positive result is shown by an improvement in ptosis (Almeida *et al*., 2008). However, there may be logistical challenges of performing the ice pack test if there is limited access to an ice pack within a clinical environment due to storage requirements. In addition, current literature may also overestimate the sensitivity and specificity of the test, with it being difficult to draw conclusions on patients who may have MG (Benatar, 2006).

### Limitations and areas for future study

Limitations of this service evaluation include its retrospective nature and its small sample size. We acknowledge that retrospective studies have limitations that one must consider when interpreting their findings. One limitation in retrospective data is the potential for incomplete data, as these are taken from a clinical report rather than a pro forma created specifically for research. The small cohort size restricts the conclusions that this service evaluation can draw. The small cohort reflects the smaller number of patients being investigated for MG in the strabismus service. In comparison, if another service within ophthalmology were assessed, such as neuro-ophthalmology or those presenting to an emergency eye clinic, a larger sample size may be obtained. Additionally, ACR testing was the first-line diagnostic test in this cohort, but further diagnostic tests for MG are available. It is also difficult to draw conclusions about mortality risk within this cohort due to the small sample size. Further studies could include larger cohorts from other areas for comparison.

Other limitations may be interobserver variation in determining eliciting MG signs, such as Cogan’s, as these require subjective interpretation. Future work could address inter-observer variation by standardising the investigation and documentation of findings for suspected MG, potentially utilising a pro forma, highlighting when escalation of further testing is required along with ensuring all suitable symptoms are discussed and clinical signs elicited. There are current patient-related outcome measures and symptom questionnaires that address the severity of MG once it has been diagnosed, but they are currently not in place to address the specific issue of diagnosis and when further investigations should take place. These include the Quantitative MG scale, MG Composite score, MG Activities of Daily Living, MG Symptoms Patient-Reported Outcome and OMG Rating Scale Questionnaire. All of these measures include ocular difficulties, which may include blurry vision, double vision, eye movements, and eyelid drooping to varying levels, but they do not include all (Carr *et al*., 2010; Regnault *et al*., 2023; Wong *et al*., 2025).

## Conclusion

In conclusion, our cohort did not identify any patients with isolated IRW who had MG, suggesting isolated IRW is not a strong indicator for MG. All patients who were diagnosed with MG were found to have additional MG characteristic symptoms or signs: variability, fatigue on elevation, Cogan’s lid twitch, MG-like fatigue, breathing difficulties and swallowing difficulties or the presence of another autoimmune condition. All patients had a weakness or restriction of two or more EOMs, with SR being the most common. Around half of the isolated IRW patients had previously had strabismus surgery before, and this may account for their isolated weakness.
